# Association of smoking and cancer with the risk of venous thromboembolism: the Scandinavian Thrombosis and Cancer cohort

**DOI:** 10.1038/s41598-021-98062-0

**Published:** 2021-09-21

**Authors:** Benedikte Paulsen, Olga V. Gran, Marianne T. Severinsen, Jens Hammerstrøm, Søren R. Kristensen, Suzanne C. Cannegieter, Hanne Skille, Anne Tjønneland, Frits R. Rosendaal, Kim Overvad, Inger Anne Næss, John-Bjarne Hansen, Sigrid K. Brækkan

**Affiliations:** 1 Thrombosis Research Center (TREC), Department of Clinical Medicine, UiT - The Arctic University of Norway, Tromsö, Norway; 2 Department of Clinical Medicine, Aalborg University, Aalborg, Denmark; 3 Department of Hematology, Aalborg University Hospital, Aalborg, Denmark; 4 Department of Cancer Research and Molecular Medicine, Norwegian University of Science and Technology, Trondheim, Norway; 5 St. Olavs Hospital, Trondheim University Hospital, Trondheim, Norway; 6 Department of Clinical Biochemistry, Aalborg University Hospital, Aalborg, Denmark; 7 Department of Clinical Epidemiology, Leiden University Medical Center, Leiden, The Netherlands; 8 Diet, Genes and Environment, Danish Cancer Society Research Center, Copenhagen, Denmark; 9 Department of Public Health, Aarhus University, Aarhus, Denmark; 10 Department of Public Health and General Practice, HUNT Research Centre, Norwegian University of Science and Technology, Levanger, Norway; 11 Division of Internal Medicine, University Hospital of North Norway, Tromsö, Norway

**Keywords:** Thromboembolism, Cancer

## Abstract

Smoking is a well-established risk factor for cancer, and cancer patients have a high risk of venous thromboembolism (VTE). Conflicting results have been reported on the association between smoking and risk of VTE, and the effect of smoking on VTE-risk in subjects with cancer is scarcely studied. We aimed to investigate the association between smoking and VTE in subjects with and without cancer in a large population-based cohort. The Scandinavian Thrombosis and Cancer (STAC) cohort included 144,952 participants followed from 1993–1997 to 2008–2012. Information on smoking habits was derived from self-administered questionnaires. Active cancer was defined as the first two years following the date of cancer diagnosis. Former smokers (n = 35,890) and those with missing information on smoking status (n = 3680) at baseline were excluded. During a mean follow up of 11 years, 10,181 participants were diagnosed with cancer, and 1611 developed incident VTE, of which 214 were cancer-related. Smoking was associated with a 50% increased risk of VTE (HR 1.49, 95% CI 1.12–1.98) in cancer patients, whereas no association was found in cancer-free subjects (HR 1.07, 95% CI 0.96–1.20). In cancer patients, the risk of VTE among smokers remained unchanged after adjustment for cancer site and metastasis. Stratified analyses showed that smoking was a risk factor for VTE among those with smoking-related and advanced cancers. In conclusion, smoking was associated with increased VTE risk in subjects with active cancer, but not in those without cancer. Our findings imply a biological interaction between cancer and smoking on the risk of VTE.

## Introduction

Venous thromboembolism (VTE), a collective term for deep vein thrombosis (DVT) and pulmonary embolism (PE), is a common disease associated with substantial morbidity and mortality^1,2^. Cancer is established as one of the leading risk factors for VTE, as cancer patients have a four- to sevenfold increased risk of VTE^3,4^, and 20–30% of all incident VTE-events are cancer-related^5^.

Cigarette smoking is recognized as a leading cause of preventable deaths in high-income countries^6^, and today, approximately one billion of the world’s population are daily smokers^7^. The harmful effects of smoking on the risk of cancer and cardiovascular disease are well established. Smoking is a risk factor for several types of cancer including lung, esophageal, laryngeal, oral cavity, renal and bladder cancers^8^.

Previous studies investigating the association between smoking and VTE have shown conflicting results. Several studies have reported no association between smoking status and risk of VTE^9–12^, whereas other studies have indicated an increased risk of VTE among current and former smokers^13^, among current smokers only^14,15^, and among heavy smokers only^16–19^. In a recent meta-analysis of cohorts with validated VTE-events, current smoking was associated with a 20% increased risk of provoked VTE^20^. This observation may suggest that the association between smoking and VTE is largely mediated through development of other comorbidities. Accordingly, heavy smoking was associated with increased risk of VTE in the Tromsø Study, but this association disappeared in cause-specific analyses where cancer and myocardial infarction were taken into account^21^.

Smoking has been shown to interact with other inherited and acquired risk factors for VTE such as prothrombotic mutations^13,22^, oral contraceptives^13^, pregnancy^23–26^, and surgery^27^. Likewise, cancer is known to act synergistically with prothrombotic mutations^28^, various blood parameters^29,30^, comorbid conditions^31^ and hematopoietic growth factors^32^ on the risk of VTE. Both smoking and cancer are known to influence the hemostatic system separately via several mechanisms including increased levels of coagulation factors and fibrinogen, impaired fibrinolysis, endothelial dysfunction and increased platelet aggregation^33,34^. It is likely to assume that coagulation abnormalities arising from two distinct acquired sources could increase the risk of VTE. However, the separate effects of smoking on the risk of VTE in subjects with and without cancer has thus far not been investigated. Therefore, using the Scandinavian Thrombosis and Cancer (STAC) cohort, a large population-based cohort with over 144,000 unique individuals, we aimed to investigate the effects of smoking on the risk of VTE in participants with and without cancer.

## Methods

### Study population

The Scandinavian Thrombosis and Cancer (STAC) cohort is comprised of three large population-based cohorts with enrolment from 1993 to 1997. The STAC cohort consists of the fourth survey of the Tromsø Study (Tromsø 4, Norway), the second Nord-Trøndelag Health Study (HUNT2, Norway) and the Diet, Cancer and Health Study (DCH, Denmark). The three individual cohorts^35–37^ and the STAC cohort^38^ have previously been described in detail. In Tromsø 4, all inhabitants ≥ 25 years living in the municipality of Tromsø were invited and 27,158 (77%) participated. In the HUNT 2 Study, all residents of the Nord-Trøndelag county ≥ 20 years were invited to take part in the survey, and 65,237 (69%) participated. In the DCH Study, inhabitants aged 50 to 64 years living in the urban areas of Copenhagen and Aarhus, without a previous cancer were invited to participate, and 57,054 (35%) attended. Study participants were followed up from the day of inclusion in the individual cohorts (1993–1997) until the end of follow-up (2007–2012). Participants with a pre-baseline cancer or VTE diagnosis were excluded before merging the cohorts, yielding a study population of 144,952 individuals in the STAC cohort. Further, subjects with missing information on smoking habits (n = 3680), as well as subjects reporting to be formers smokers at baseline (n = 35,890) were excluded. The study cohort, therefore, consisted of 105,382 participants aged 19 to 101 years.

The regional committees for research ethics in Norway and Denmark approved the respective cohort studies and the collaboration study. All participants in the cohorts provided informed written consent before inclusion. All methods were performed in accordance with the relevant guidelines and regulations.

We chose to merge the three cohorts and perform individual data analysis because the structure of the individual cohorts, the assessment of outcomes (VTE registrations), and the cancer registries in Norway and Denmark were quite homogenous. Harmonization of the data before merging and close control of the models further reduced heterogeneity, and the pooled individual data approach would yield higher statistical power than a meta-analysis approach.

### Baseline measurements

Baseline information on lifestyle and cardiovascular risk factors was collected by means of physical examination and self-administered questionnaires. Body weight and height were measured in subjects wearing light clothing and no shoes. Body mass index (BMI) was calculated as the weight in kilograms (kg) divided by the square of the height in meters (m). Information regarding self-reported diabetes, level of education (basic school, high school, and university/college) and lifestyle factors, including smoking habits, physical activity level and alcohol consumption was collected by self-administered questionnaires. Alcohol consumption was defined as the reported average number of units per week. Information on smoking habits was also collected from the questionnaires. Smoking status was categorized into never smokers and current smokers. Current smokers were further dichotomized into categories according to amount of daily smoking; < 15 cigarettes per day; and ≥ 15 cigarettes per day.

### Identification of cancer

In the STAC cohort, all first lifetime diagnoses of cancer during follow-up were identified by linkage to the Danish Cancer Registry and the Cancer Registry of Norway. The unique national civil registration number assigned to all people residing in the Nordic countries was used for the linkage^38^. The cancer registries receive information from general practitioners, hospital doctors, pathological laboratories, and death certificates^39,40^, and provide information on date of cancer diagnosis, primary site of disease (ICD10 codes C00-96), tumor histology (ICO-3) and metastasis (localized, regional, distant, or unknown). Both cancer registries are considered high quality, and evaluations have reported a 98.8% completeness in Norway and 95–98% in Denmark, with histological verification of 94% and 93% diagnoses, respectively^39,40^.

### Identification and validation of venous thromboembolism

All symptomatic, objectively confirmed, first lifetime VTE events that occurred during follow-up were recorded after thorough identification and validation. Potential cases of VTE were identified by searching discharge diagnosis registries, radiology procedure registries, cause of death registries and autopsy registries at each of the hospitals covering the study sites, as previously described^38^. The medical records of each potential VTE case were reviewed by trained medical professionals, and adjudicated using similar confirmatory criteria in each cohort. A VTE event was only included if traditional signs and symptoms of DVT and/or PE were noted in the medical records, objective diagnostic tests confirmed the diagnosis of VTE (e.g. compression ultrasonography, venography, spiral computed tomography, perfusion-ventilation scan, pulmonary angiography, or autopsy) and appropriate treatment was initiated. In Tromsø and DCH the autopsy registries were also searched. Events identified through the autopsy registry were included if VTE was noted as the cause of death or as a significant condition contributing to death on the death certificate. Detailed description regarding VTE identification and validation in each of the three cohorts has been published previously^10,15,41^. VTE events were classified as either a DVT or PE, and if the two events occurred concurrently, the event was registered as a PE.

### Statistical analysis

Statistical analysis was performed using STATA version 14 (Stata Corporation LP, College Station, Texas, USA). Participants were followed from the date of inclusion in one of the cohorts until the date of an incident VTE, emigration, death or end of follow-up (Dec 31, 2012 in Tromsø, December 31, 2007 in HUNT 2, and April 30, 2008 in DCH). Cancer was entered as a time-varying co-variate in the analysis. Thus, participants who developed cancer during follow-up contributed with person-time to the “no cancer” category from the date of inclusion until the date of cancer diagnosis, and thereafter in the “cancer” category until two years after the cancer diagnosis (active cancer time). VTE-events occurring during this two-year period were considered to be cancer-related. Participants who were alive and VTE-free at the end of the active cancer period were censored, as extending the observation period could potentially result in dilution of the results due to lack of information on cancer remission and relapses during follow-up.

The median follow-up time with inter quartile range (IQR) was calculated using descriptive statistics. Incidence rates (IR) were calculated by dividing the total number of events by the person-time at risk and expressed as number of events per 1000 person-years. Cancer-exposed person-time was calculated from the date of cancer diagnosis and onwards (until VTE, migration, death or censoring two years after cancer diagnosis, as described above). Cox proportional hazard regression models were used to estimate hazard ratios (HR) with 95% confidence intervals (CI) across categories of baseline smoking status in participants with and without cancer. The risk was assessed in two adjustment models. Model 1 included age, sex and BMI. Model 2 was additionally adjusted for cancer site and metastasis. The proportional hazards assumption was tested using Schoenfeld residuals.

To further investigate whether an effect on VTE in smokers with cancer could be explained by a higher proportion of thrombogenic cancer types in smokers, we stratified our data into smoking-related cancer sites and cancer sites that was not related to smoking. The classification smoking-related cancer was based on the International Agency for Research on Cancer (IARC) monograph about the causal association between tobacco use and cancer^8^. Smoking-related cancers were defined as lung, urinary tract (urether and bladder), kidney, renal pelvis, ENT (= ear, nose and throat; nasal- and oral cavity, sinuses, middle ear, pharynx), larynx, lung, oesophagus, stomach, colorectal, pancreas and liver. Of note, acute myeloid leukaemia (AML) is related to smoking, but as our data lacked stratification on type of leukaemia, AML could not be included in the definition of smoking-related cancer.

We also wanted to explore whether the relationship between smoking and VTE could be explained by more advanced cancers in smokers. We therefore stratified our analyses according to the degree of metastasis (local, regional, distant and unknown).

For the clinical interpretation of this paper, we investigated the risk of VTE according to smoking status in patients with overt cancer (i.e., from the date of cancer diagnosis). However, since VTE can be the first sign of an underlying (occult) malignancy, we also performed sensitivity analyses where we defined active cancer as the period 6 months before to 2 years after the VTE diagnosis and rerun the main analysis. Moreover, since VTE may not be related to cancer in those successfully cured after a short treatment period, we performed a second sensitivity analysis restricted to cancer patients with distant metastasis (i.e., non-curable disease) followed throughout the entire study period.

## Results

Characteristics, assessed at cohort enrolment, in participants of the entire cohort (n = 105,382) and those who developed cancer during follow-up (n = 10,181) are presented in Table 1. The mean age at inclusion was 51 years and 44% of the participants were men. As expected, those who developed cancer were generally older (58 years vs. 51 years) and more often smokers (55% vs 46%) when compared with the entire cohort.

**Table 1:** Baseline characteristics of the entire cohort and subjects with active cancer.

	Entire cohort			Cancer		
	All	Men	Women	All	Men	Women
n	105 382	46,756	58,626	10,181	4782	5399
Age, (years, mean ± SD)	51 ± 14	50 ± 13	51 ± 14	58 ± 10	59 ± 10	58 ± 10
BMI, (kg/m^2^, mean ± SD)	25.8 ± 4.1	26.1 ± 3.5	25.6 ± 4.5	26.1 ± 4.3	26.2 ± 3.7	25.9 ± 4.7
Daily smoking, % (n)	45.9 (48 341)	50.9 (23 833)	41.8 (24 508)	55.0 (5 602)	63.3 (3 028)	47.7 (2 574)
Diabetes, % (n)	2.2 (2 323)	2.3 (1 086)	2.1 (1 237)	3.6 (361)	3.7 (187)	3.3 (183)
Use of antihypertensives, % (n)	9.7 (10 243)	8.2 (3 843)	10.9 (6 400)	14.5 (1 453)	12.9 (619)	15.4 (834)
Alcohol intake (units/week)	5.7 ± 9.8	7.9 ± 12	6.7 ± 7.0	7.4 ± 12	10.3 ± 15	4.9 ± 7.8
Education level						
Basic School, % (n)	27.1 (28 628)	22.1 (10 325)	31.4 (18 303)	34.6 (3 468)	29.9 (1 433)	37.7 (2 035)
High School, % (n)	48.8 (51 450)	48.9 (22 855)	49.1 (28 595)	46.1 (4 624)	44.3 (2 120)	46.4 (2 504)
Collage/University, % (n)	21.5 (22 729)	27.5 (12 573)	17.4 (10 153)	17.2 (1 731)	22.5 (1 079)	12.1 (652)
Physical activity > 1 h/week, % (n)	27.4 (28 897)	32.3 (15 229)	23.3 (13 668)	21.4 (2 145)	24.2 (1 160)	18.2 (985)

Values are numbers or percentages with numbers or means ± SD in parenthesis.

Active cancer: period from six months before a cancer diagnosis until two years after.

*BMI* body mass index, Daily smoking indicates smoking at the time of enrollment; diabetes mellitus;

During a median follow-up of 11.6 years (IQR: 10.6–12.3), 1611 developed an incident VTE, of which 214 were related to active cancer. The incidence rate (IR) of VTE was 1.4 per 1000 person-years (PY) in the total cohort, and 14.1 per 1000 PY in patients with active cancer. Accordingly, the risk of VTE was eightfold (HR 8.27, 95% CI 7.14–9.57) higher in patients with active cancer when compared to cancer-free subjects after adjustment for age and sex.

At the time of cancer diagnosis, 35.6% of cancers were localized, 24.3% had regional metastases, 15.4% had distant metastases, while the remaining 24.7% either had missing information on metastasis or used non-traditional cancer staging (i.e., haematological cancers). Breast, prostate and colorectal cancers were the most frequent cancers in the study population. The distribution of cancer site and metastasis at cancer diagnosis among never smokers and smokers is presented in Table 2. Smokers tended to present with more advanced disease at cancer diagnosis, as 19.3% of the smokers and 10.5% of the never smokers had distant metastases at the time of cancer diagnosis. Among smokers, lung cancer was the most frequent cancer site accounting for 21.5% of the cancers, while the corresponding proportion was only 2.2% among never smokers. In addition, urological (5.8% vs. 3.9%) and upper gastrointestinal (7.9% vs. 5.2%) cancers were more frequent among smokers than among never smokers.

**Table 2:** Distribution of cancer site and metastasis in smokers and never-smokers.

	Never-smokers	Smokers
Subjects, n	4 579	5 602
Age (years), mean ± SD	60 ± 11	58 ± 9
Cancer site		
ENT, % (n)	1.3 (58)	2.6 (143)
Upper GI*, % (n)	3.9 (177)	5.8 (326)
Colorectal, % (n)	16.2 (742)	11.4 (638)
Pancreas, % (n)	2.3 (107)	3.1 (176)
Lung, % (n)	2.2 (102)	21.5 (1205)
Melanoma, % (n)	5.6 (257)	2.6 (144)
Gynaecological, % (n)	9.2 (421)	4.4 (245)
Breast, % (n)	20.9 (961)	12.7 (711)
Urological, % (n)	5.2 (238)	7.9 (442)
Prostate, % (n)	15.3 (700)	13.0 (726)
CNS, % (n)	3.9 (180)	2.7 (151)
Hem/lymph, % (n)	9.0 (411)	6.1 (341)
Other, % (n)	4.4 (200)	5.2 (294)
Missing	0.5 (25)	0.3 (15)
Metastasis		
Localized, % (n)	38.5 (1 762)	33.3 (1 865)
Regional, % (n)	24.5 (1 120)	24.2 (1 358)
Distant metastasis, % (n)	10.5 (483)	19.3 (1 082)
Unknown, % (n)	26.5 (1214)	23.2 (1297)

Values are percentages with numbers in parenthesis.

*Includes oesophagus, stomach, small intestine, liver, gallbladder and biliary tract cancers. *ENT* Ear, nose and throat, *CNS* central nervous system.

IRs and HRs of VTE according to smoking status in participants with and without cancer are presented in Table 3. In cancer-free subjects, smoking was not associated with increased risk of VTE (HR 1.07, 95% CI 0.96–1.20). In patients with active cancer, smokers had 50% higher VTE risk than never-smokers (HR 1.49, 95% CI 1.12–1.98), and further adjustment for cancer site and metastasis had negligible impact on the risk estimate (HR 1.43, 95% CI 1.07–1.91). Stratification on cancer sites particularly related to smoking, revealed that smoking was associated with a 1.3-fold higher risk of VTE among those who developed cancer at sites that were typically related to smoking (HR 1.32, 95% CI 0.89–1.96). In those who developed cancer types not typically related to smoking, current smoking was not associated with increased risk of VTE (Table 4). Moreover, smoking was associated with increased VTE risk in cancer patients with regional- (HR 1.53, 95% CI 0.89–2.64) and distant metastasis (HR 1.69, 95% CI 0.97–2.96) (Table 5).

**Table 3:** Incidence rates and hazard ratios of venous thromboembolism (VTE) according to cancer and smoking status.

	VTE	IR (95% CI)	Model 1 HR (95%CI)	Model 2 HR (95%CI)
**VTE**				
No cancer				
Never smoker	814	1.3 (1.2–1.4)	Ref	*–*
Current smoker	583	1.1 (1.0–1.2)	1.07 (0.96–1.20)	*–*
Cancer				
Never smoker	87	12.6 (10.2–15.6)	Ref	Ref
Current smoker	127	17.3 (14.5–20.6)	1.49 (1.12–1.98)	1.43 (1.07–1.91)
**DVT**				
No cancer				
Never smoker	465	0.7 (0.6–0.8)	Ref	*–*
Current smoker	349	0.6 (0.5–0.7)	1.11 (0.96–1.28)	*–*
Cancer				
Never smoker	53	7.7 (5.9–10.1)	Ref	Ref
Current smoker	76	10.4 (8.2–13.0)	1.50 (1.04–2.15)	1.50 (1.03–2.21)
**PE**				
No cancer				
Never smoker	345	0.5 (0.5–0.6)	Ref	*–*
Current smoker	223	0.4 (0.3–0.5)	0.98 (0.82–1.17)	*–*
Cancer				
Never smoker	32	4.7 (3.3–6.6)	Ref	Ref
Current smoker	47	6.4 (4.8–8.5)	1.47 (0.92–2.34)	1.39 (0.86–2.26)

*CI* confidence intervals, *HR* hazard ratio, *IR* incidence rates presented per 1000 person-years; Model 1: Adjusted for age, sex and BMI; Model 2: Adjusted for age, sex, BMI and cancer site and metastasis.

Cancer was entered as a time-varying covariate in our models. Cancer-exposed person-time was calculated from the date of cancer diagnosis until a VTE, migration, death or end of the active cancer period (two years after cancer diagnosis).

**Table 4:** Incidence rates and hazard ratios of venous thromboembolism (VTE) according to smoking status in patients with and without cancer types that are classified as smoking-related.

	VTE	IR (95% CI)	Model 1 HR (95% CI)	Model 2 HR (95% CI)
**Patients with cancer types related to smoking**				
Never smoker	39	21.3 (15.6–29.1)	Ref	Ref
Current smoker	96	27.9 (22.8–34.0)	1.36 (0.92–2.02)	1.32 (0.89–1.96)
**Patients with cancer types not related to smoking**				
Never smoker	48	9.5 (7.2–12.6)	Ref	Ref
Current smoker	31	8.0 (5.6–11.4)	0.96 (0.60–1.52)	0.94 (0.59–1.50)

Cancer types related to smoking were defined according to the International Agency for Research on Cancer (IARC), and included lung, urinary tract, kidney, renal pelvis, ENT (ear, nose and throat), larynx, lung, oesophagus, stomach, colorectal, pancreas, and liver.

*CI* confidence intervals, *HR* hazard ratios, *IR* incidence rates presented per 1000 person-years.

Model 1: Adjusted for age, sex and BMI; Model 2: adjusted for age, sex, BMI and metastasis.

**Table 5:** Incidence rates and hazard ratios of venous thromboembolism (VTE) according to smoking status in cancer patients with localized, regional and distant metastasis, respectively.

	VTE	IR (95% CI)	Model 1 HR (95% CI)	Model 2 HR (95% CI)
**Cancer patients with localized metastasis**				
Never smoker	22	7.3 (4.8–11.1)	Ref	Ref
Current smoker	21	7.0 (4.5–10.7)	1.04 (0.56–1.94)	1.09 (0.58–2.03)
**Cancer patients with regional metastasis**				
Never smoker	22	13.4 (8.8–20.4)	Ref	Ref
Current smoker	38	21.7 (15.8–29.8)	1.53 (0.89–2.64)	1.50 (0.87–2.59)
**Cancer patients with distant metastasis**				
Never smoker	19	40.6 (25.9–63.6)	Ref	Ref
Current smoker	47	61.3 (46.1–81.6)	1.69 (0.97–2.96)	1.66 (0.94–2.93)
**Cancer patients with unknown metastasis**				
Never smoker	24	13.6 (9.1–20.1)	Ref	Ref
Current smoker	21	11.7 (7.6–17.9)	1.07 (0.57–1.98)	1.08 (0.58–2.03)

In cancer-free subjects, the VTE risk according to smoking was 0.93 (95% CI 0.80–1.10) in men and 1.22 (95% CI 1.04–1.42) in women (Supplementary Table 1). In cancer patients, the risk estimate for VTE according to smoking status was 1.68 in women (95% CI 1.15–2.45) and 1.30 in men (95% CI 0.85–2.00), and persisted after multivariable adjustment for age, sex, BMI, cancer site and metastasis. Analyses of the daily quantity of cigarettes smoked (never smoker, < 15 cigarettes daily and ≥ 15 cigarettes daily) showed that an increase in quantity of daily smoking (at a threshold of 15 cigarettes daily) had little influence on the risk of VTE (Supplementary Table 2). Our sensitivity analyses showed that smoking remained a risk factor for VTE in cancer patients (HR 1.30, 95% CI 1.01–1.67) when the active cancer period was extended to include the period 6 months before diagnosis (i.e., occult cancer), and in analyses restricted to those with distant metastasis with unlimited follow-up (HR 2.05, 95% CI 1.21–3.49) (Supplementary table 3).

## Discussion

In the present study, current smoking was associated with 50% increased risk of VTE in patients with active cancer. In contrast, smoking was not associated with VTE in cancer-free subjects. In cancer patients, the risk of VTE among current smokers remained unchanged after adjustments for cancer site and metastasis, suggesting that the effect was not explained by more advanced cancers in smokers than non-smokers. Moreover, smoking was associated with increased risk of VTE in those with cancer types that are typically related to smoking and in patients with regional- and distant metastasis.

As smoking increases blood coagulability and impairs endothelial function and fibrinolysis^33^, it is etiologically sound to expect smoking to increase the risk of VTE. In our study, however, current smoking was not associated with an increased risk of VTE in cancer-free subjects. Accordingly, large population cohorts, such as the HUNT2 Study^12^, the Physicians' Health Study^9^, the Framingham Study^42^, and the Longitudinal Investigation of Venous Thromboembolism (LITE) Study^11^, also reported no increased risk of VTE related to smoking status. However, other studies have reported an increased risk of VTE among current and formers smokers^13^, among current smokers only^14,15^, and among heavy smokers only^16–19^. Furthermore, some studies have reported an association only between smoking and provoked VTE^21,43^. The diverging results could be attributed to differences in the distribution of patients with cancer and other co-morbid conditions associated with VTE. Taken together, the studies suggest that smoking alone is not sufficient to develop VTE, but when combined with some co-morbidities, such as cancer, smoking could result in a total risk factor level that exceeds the patient’s thrombosis threshold.

A few previous studies have reported an increased VTE risk in smokers with cancer. In a prospective observational study of 381 gastrointestinal or breast cancer patients receiving adjuvant chemotherapy, smoking (former and current) was associated with a twofold increased risk of VTE^44^. In the Iowa Women’s Health Study, a cohort study of older women, the observed association between current and former smoking and risk of VTE was mainly driven by cancer-related VTEs among smokers^43^. Further, results from the Tromsø Study, showed that heavy smoking (> 20 pack years) was associated with a 1.8-fold increased risk of provoked VTE^21^, but the apparent association disappeared when intermediate development of cancer and myocardial infarction was taken into consideration. Our findings suggest that cancer patients who smoke have a higher risk of VTE than those who do not smoke.

A possible explanation for the combined effect of smoking and active cancer could be that smoking infer more advanced cancer (e.g. distant metastasis) and prothrombotic cancer types (e.g. lung cancer). Accordingly, smoking was associated with prothrombotic cancer types (e.g. lung, upper gastrointestinal and urological) and the proportion with distant metastasis was higher in smokers than in non-smokers in our study. However, the higher risk of VTE in cancer patients who smoked remained unchanged after adjustments for these factors, and smoking was also a risk factor for VTE in analyses restricted to cancer sites that were typically related to smoking. Moreover, smoking was a risk factor for VTE also in analyses restricted to those with regional- and distant metastasis. This implies that the increased risk of VTE by smoking in cancer patients was not explained by more advanced cancer and prothrombotic cancer types in smokers. However, it is likely that more prothrombotic cancers (i.e. smoking-related cancers) contributes to exceeding the thrombosis threshold when combined with smoking. This could potentially explain why no association was observed in patients with cancers that were not related to smoking and those with localized cancer.

Smoking has previously been reported to interact with other acquired and inherited risk factors for VTE. Using the MEGA Study, Pomp and colleagues reported that both former and current smoking in combination with oral contraceptive use synergistically increased the risk of VTE in cancer-free subjects^13^. In a prospective study of 56,014 participants in the DCH Study, heavy smoking and the non-O blood type demonstrated a positive interaction on the risk of VTE, with the joint effects being responsible for an additional 30.5 VTE events per 100,000 person-years^45^. In addition, other studies have reported on the interaction between smoking and several risk factors for VTE including prothrombotic mutations^13,22^, pregnancy^23–26^, and surgery^27^. Furthermore, previous studies have demonstrated that cancer also acts synergistically with several inherited and acquired risk factors for VTE^28–32^. In the present study, smoking was associated with VTE only in cancer patients, which indicates a biological interaction between smoking and cancer on the VTE risk. Smoking results in widespread systemic effects in the body causing changes to both hemostatic and inflammatory processes^33,34^, and the procoagulant nature of cancer is well-established^34^. Thus, it is likely to assume that a biological interaction between smoking and cancer on risk of VTE could be mediated via procoagulant changes arising from two sources.

The increased risk of VTE among cancer patients who smoked encourage recommendation of smoking cessation in order to avoid VTE in cancer patients. Current risk prediction scores for cancer-related VTE are based on clinical (e.g. cancer site, BMI) and laboratory (e.g. hemoglobin levels, platelet and leukocyte counts) parameters, and are thought to have limited clinical utility as they have a poor ability to distinguish between patients at high and low risk of VTE^46^. Our finding of smoking as a risk factor for VTE in cancer may suggest to include smoking status in the development of future prediction models of VTE risk, particularly in patients with cancer types that are likely related to smoking.

The main strength of our study was the large sample size and comprehensive information at baseline, which enabled etiological analysis where potential confounders could be accounted for. The completeness and validity of the cancer registries in Norway and Denmark is very high^39,40^. The study also had some limitations. Assessment of tobacco exposure was obtained at baseline inclusion in the respective cohorts, and changes in smoking habits could potentially result in misclassification of exposure during follow-up. However, data from the DCH five-year follow-up questionnaires showed that 80% of smokers maintained the same smoking status^22^, and, in all three cohorts, changes in smoking status in the population were towards lower tobacco use^36,47^. In our statistical models, we assumed that smokers diagnosed with cancer continued to smoke. However, it is likely that some would quit smoking after being diagnosed with cancer. Changes in smoking status would likely result in an underestimation of the association between smoking and VTE due non-differential misclassification and, consequently, regression dilution bias. Unfortunately, we did not have information on type of cancer treatment. However, treatment choice would probably not be influence by smoking status, but rather be dependent on the type and advancement of the cancer. In stratified analyses, we showed that smoking was associated with risk of VTE both in those with cancer sites typically related to smoking, and when restricted to those with regional or distant metastasis.

In conclusion, current smoking was associated with 50% increased risk of VTE in subjects with cancer, whereas smoking was not associated with VTE risk in subjects without cancer. The predictive impact of smoking on VTE in cancer patients should be investigated in future studies.

## Supplementary Information

Supplementary Information.

## Data Availability

The data can be made available upon request to the steering committees of the different study cohorts.
