# Supplementary material for: Association of smoking and cancer with the risk of venous thromboembolism: the Scandinavian Thrombosis and Cancer cohort
Source: Sci Rep. 2021 Sep 21;11:18752. doi: 10.1038/s41598-021-98062-0 (PMC8455552; doi:10.1038/s41598-021-98062-0)
Supplement: Supplementary file 1 — Supplementary Information. [file 41598_2021_98062_MOESM1_ESM.docx]

**Supplementary Table 1:** Incidence rates (IR) and hazard ratios (HR) for venous thromboembolism (VTE) across categories of smoking and cancer status for men and women.

|  | **VTE** | **IR (95%CI)** | **Model 1**  **HR (95%CI)** | **Model 2**  **HR (95%CI)** |
| --- | --- | --- | --- | --- |
| **Men** |  |  |  |  |
| **No cancer** |  |  |  |  |
| Never smoker | 311 | 1.2 (1.0-1.3) | Ref. | - |
| Current smoker | 305 | 1.1 (1.0-1.3) | 0.94 (0.80-1.10) | - |
| **Cancer** |  |  |  |  |
| Never smoker | 32 | 12.4 (8.7-17.6) | Ref | Ref. |
| Current smoker | 62 | 16.4 (12.8-21.0) | 1.30 (0.85-2.00) | 1.25 (0.81-1.93) |
| **Women** |  |  |  |  |
| **No cancer** |  |  |  |  |
| Never smoker | 503 | 1.3 (1.2-1.4) | Ref. | - |
| Current smoker | 278 | 1.0 (0.9-1.1) | 1.22 (1.04-1.42) | - |
| **Cancer** |  |  |  |  |
| Never smoker | 55 | 12.8 (9.7-16.6) | Ref. | Ref. |
| Current smoker | 65 | 18.3 (14.4-23.3) | 1.68 (1.15-2.45) | 1.60 (1.09-2.34) |

CI, confidence intervals; HR, hazard ratio; IR, incidence rates presented per 1000 person-years

Model 1: Adjusted for age, sex and BMI; Model 2: Adjusted for age, sex, BMI, cancer site and metastasis.

**Supplementary Table 2:** Incidence rates (IRs) and hazard ratios (HRs) with 95% confidence intervals for venous thromboembolism (VTE) according to categories of smoking status stratified by cancer: The STAC-cohort

|  | **VTE** | **IR (95% CI)** | | **Model 1**  **HR (95% CI)** | | **Model 2**  **HR (95% CI)** |
| --- | --- | --- | --- | --- | --- | --- |
| **No cancer** | | |  | |  |  |
| Never smoker | 814 | 1.3 (1.2-1.4) | | Ref. | | - |
| <15 cigarettes daily | 292 | 1.0 (0.8-1.1) | | 1.01 (0.89-1.16) | | - |
| ≥15 cigarettes daily | 291 | 1.2 (1.1-1.3) | | 1.13 (0.98-1.30) | | - |
| **Active Cancer** | | |  | |  |  |
| Never smoker | 87 | 12.6 (10.2-15.6) | | Ref. | | Ref. |
| <15 cigarettes daily | 65 | 18.2 (14.2-23.1) | | 1.52 (1.10-2.12) | | 1.53 (1.10-2.13) |
| ≥15 cigarettes daily | 62 | 16.5 (12.9-21.2) | | 1.44 (1.03-2.03) | | 1.33 (0.94-1.88) |

CI, confidence intervals; HR, hazard ratio; IR, incidence rates presented per 1000 person-years

Model 1: Adjusted for age, sex and body mass index (BMI); Model 2: Adjusted for age, sex, BMI and cancer site and metastasis.

**Supplementary Table 3:** Incidence rates and hazard ratios of venous thromboembolism (VTE) according to cancer and smoking status in sensitivity analyses with (i) active cancer was defined as 6 months before to 2 years after cancer diagnosis, and (ii) analyses restricted to cancer patients with distant metastasis and full follow-up after cancer diagnosis.

|  | **VTE** | | **IR (95% CI)** | | **Model 1**  **HR (95%CI)** | | **Model 2**  **HR (95%CI)** | |  |
| --- | --- | --- | --- | --- | --- | --- | --- | --- | --- |
| **Active cancer defined as 6 months before to 2 years after cancer diagnosis** | | | | | | | | |  |
| **No cancer** |  | |  | |  | |  | |  |
| Never smoker | 783 | | 1.2 (1.1-1.3) | | Ref. | | - | |  |
| Current smoker | 553 | | 1.0 (0.9-1.1) | | 1.05 (0.94-1.18) | | - | |  |
| **Cancer** |  | |  | |  | |  | |  |
| Never smoker | 118 | | 12.9 (10.7-15.4) | | Ref | | Ref. | |  |
| Current smoker | 157 | | 15.5 (13.2-18.1) | | 1.34 (1.05-1.73) | | 1.30 (1.01-1.67) | |  |
| **Restricted to cancer patients with distant metastasis at diagnosis and full follow-up** | | | | | | | | | |
| **Distant metastasis** | |  | |  | |  | |  | |
| Never smoker | | 22 | | 27.3 (18.1-41.5) | | Ref | | Ref. | |
| Current smoker | | 52 | | 51.6 (39.3-67.8) | | 2.16 (1.28-3.63) | | 2.05 (1.21-3.49) | |

Model 1: Adjusted for age, sex and body mass index (BMI); Model 2: Adjusted for age, sex, BMI and cancer site and metastasis (not adjusted for metastasis in the analysis restricted to metastatic cancer).
